# Hallucinations in schizophrenia and Parkinson’s disease: an analysis of sensory modalities involved and the repercussion on patients

**DOI:** 10.1038/srep38152

**Published:** 2016-12-01

**Authors:** P. M. Llorca, B. Pereira, R. Jardri, I. Chereau-Boudet, G. Brousse, D. Misdrahi, G. Fénelon, A.-M. Tronche, R. Schwan, C. Lançon, A. Marques, M. Ulla, P. Derost, B. Debilly, F. Durif, I. de Chazeron

**Affiliations:** 1 CHU Clermont-Ferrand, Psychiatry B, Clermont-Ferrand, F-63003, France; 2 Univ Clermont 1, UFR Medecine, EA7280, Clermont-Ferrand, F-63001, France; 3 CHU Clermont-Ferrand, Biostatistics unit (DRCI), Clermont-Ferrand, F-63003, France; 4 Hôpital Fontan, CHRU de Lille, F-59000, Lille, France; 5 Laboratoire de Sciences Cognitives & Affectives (SCA-Lab), UMR CNRS 9193, Université de Lille & CURE, France; 6 Pôle de Psychiatrie Adulte, CH Charles Perrens; cs 81285, 33000 Bordeaux cedex, France; 7 CNRS UMR 5287-INCIA-“Neuroimagerie et cognition humaine”, Université Bordeaux 2, Bordeaux, France; 8 AP-HP, Groupe Hospitalier Henri-Mondor, Service de neurologie, Créteil, France; 9 INSERM U955, Equipe 1, Institut Mondor de Recherche Biomédicale, Créteil, France; 10 Ecole Normale Supérieure, Institut d’Etudes Cognitives, Paris, France; 11 Pôle hospitalier universitaire de psychiatrie du Grand Nancy, CPN Chef de Service Maison des Addictions, CHU de Nancy, France; 12 Department of Psychiatry/Department of Addiction, Sainte-Marguerite University Hospital, 13009 Marseille, France; 13 EA 3279-Public Health, Chronic Disease, and Quality of Life Research Unit, Aix-Marseille University, 13005 Marseille, France; 14 CHU Clermont-Ferrand, Neurology A, Clermont-Ferrand, F-63003, France

## Abstract

Hallucinations have been described in various clinical populations, but they are neither disorder nor disease specific. In schizophrenia patients, hallucinations are hallmark symptoms and auditory ones are described as the more frequent. In Parkinson’s disease, the descriptions of hallucination modalities are sparse, but the hallucinations do tend to have less negative consequences. Our study aims to explore the phenomenology of hallucinations in both hallucinating schizophrenia patients and Parkinson’s disease patients using the *Psycho-Sensory hAllucinations Scale* (PSAS). The main objective is to describe the phenomena of these clinical symptoms in those two specific populations. Each hallucinatory sensory modality significantly differed between Parkinson’s disease and schizophrenia patients. Auditory, olfactory/gustatory and cœnesthetic hallucinations were more frequent in schizophrenia than visual hallucinations. The guardian angel item, usually not explored in schizophrenia, was described by 46% of these patients. The combination of auditory and visual hallucinations was the most frequent for both Parkinson’s disease and schizophrenia. The repercussion index summing characteristics of each hallucination (frequency, duration, negative aspects, conviction, impact, control and sound intensity) was always higher for schizophrenia. A broader view including widespread characteristics and interdisciplinary works must be encouraged to better understand the complexity of the process involved in hallucinations.

Hallucinations have been defined by the Diagnostic and Statistical Manual of Mental Disorders fifth edition (DSM-5)^1^ as: “*perception-like experiences that occur without an external stimulus. They are vivid and clear, with the full force and impact of normal perceptions, and not under voluntary control. They may occur in any sensory modality, but auditory hallucinations are the most common in schizophrenia and related disorders. Auditory hallucinations are usually experienced as voices, whether familiar or unfamiliar, that are perceived as distinct from the individual’s own thoughts. The hallucinations must occur in the context of a clear sensorium; those that occur while falling asleep (hypnagogic) or waking up (hypnopompic) are considered to be within the range of normal experience. Hallucinations may be a normal part of religious experience in certain cultural contexts” (Schizophrenia spectrum and other psychotic disorders* section, p 87–88)^1^. However, this definition is somewhat restrictive for schizophrenia, as it is weak in terms of phenomenological aspects and specifically focused on auditory experiences. Despite the fact that hallucinations are present in the clinical criteria of various disorders (e.g. schizoaffective disorder, substance/medication-induced psychotic disorder, psychotic disorder due to other medical conditions, bipolar and related disorder, depressive disorder, anxiety disorder, etc), there is no other precise description taking into account their specificity related to the clinical context in DSM-5.

As underlined by Lowe^2^, “*the variety in the manners in which hallucinations have been defined does not imply that any given definition is invalid, but it does confirm that hallucinations are complex phenomena, whose investigation almost certainly required multi-dimensional research designs and multiple initial criteria”*.

Hallucinations must be considered as heterogeneous experiences, involving a wide variety of modalities and types including auditory, verbal, visual, olfactory, cenesthetic, gustatory and also multi-modal expression (hallucinations occurring simultaneously in more than one modality). If auditory hallucinations are the most frequently described and considered to be the most prevalent, especially in schizophrenia, other modalities or multi-modal expressions are probably underreported and more common than traditionally suggested^3^.

In a phenomenological perspective, auditory hallucinations can be better described using acoustic and linguistic properties, frequency, control, inner- vs outer-localization, content, personification, appraisals and change over time^4^. Perceptual qualities, temporal aspects, content, reality, sense of control, onset and triggers, reactions, beliefs and appraisals would help to describe visual hallucinations^5^.

In the last years, complementary approaches to the DSM have been raised. This is notably the case for the NIMH Research Domain Criteria (RDoC), which consider psychopathology in terms of maladaptive extremes along a continuum of normal functioning. Research Domain Criteria were judged optimal to promote a translational approach and encourage studying a dimension of interest in different groups, remaining “*agnostic with regard to diagnosis*”^6^. Recent works^6^,^7^ proposed that such a framework could be used to explore the complexity of auditory and visual hallucinations.

Hallucinations have been described in various clinical populations, but they are neither disorder nor disease specific. They are also frequent in non-clinical populations^4^, and an important interest has been developed for voice-hearing in the general population^8^.

In schizophrenia (SCZ) patients, hallucinations can be observed in any of the sensory modalities. In 59% of the cases they are auditory in nature, and in 27% of those cases visual hallucinations are also experienced at some point^5^. Other types of hallucinations are less prevalent.

In the course of Parkinson’s disease (PD), hallucinations occur in approximately 30 to 60% of the subjects^9^. They are frequently considered to be visual in nature, with prevalence of this modality ranging from 22 to 38%^10^, and are less frequently auditory (8% prevalence) according to Inzelberg *et al*.^11^. Olfactory hallucinations have been described in 10% of PD patients^12^. More specifically, in this population “*presence hallucinations*” include the feeling of another person being present mostly to the side or behind the subject^13^. Multimodal experiences have been reported in up to 30% of cases^14^. An important aspect is that hallucinations in PD seem to have less negative valence and less impact on patient quality of life compared to SCZ patients^12^.

The assessment of hallucinations relies on different tools to evaluate each modality and integrate various phenomenological characteristics^3^. We recently developed a multimodal hetero-evaluation scale (i.e. the *Psycho-Sensory hAllucinations Scale*: PSAS) that includes four domains (auditory, visual, olfactory/gustatory, and coenesthetic modalities) and one specific item ‘guardian angel’ defined as the “*feeling of presence to the vivid sensation that somebody is present nearby, when no one is actually there, in the absence of sensory clues revealing a presence*”, to describe the “*presence hallucinations*”, previously mentioned. We validated this scale in different populations of hallucinating patients suffering from SCZ and PD. A dimensional analysis confirmed a four-factor structure in PD patients including a first factor grouping olfactory/gustatory hallucinations and coenesthetic hallucinations, a second factor with auditory hallucinations, a third one defined by visual hallucinations and a forth one with the ‘guardian angel’ item. In patients with SCZ, a three-factor solution was confirmed, including a first factor gathering auditory, gustatory and olfactory hallucinations, a second one including mainly visual hallucinations and a third one grouping “guardian-angel” and coenesthetic hallucinations^15^.

This study aimed to explore the phenomenology of hallucinations in SCZ and PD patients using the PSAS. The objectives were i) to describe the phenomenology of these clinical symptoms in those two specific populations, and ii) to compare their specificity in those two groups.

## Methods

### Procedure

We performed a multicenter study involving five psychiatric departments and two neurological departments in France during one year. All consecutive patients were screened for study participation in order to reduce selection bias. Hallucinating patients with a diagnosis of SCZ or PD were included consecutively and were evaluated during one session by investigators.

### Evaluation tool

Hallucinations were assessed using the PSAS. It includes four domains related to the five sensory modalities (auditory, visual, olfactory/gustatory, and coenesthetic) but also another domain: ‘guardian angel’. This scale was found to have good internal consistency and good inter-rater reliability (i.e. for internal consistency Kuder–Richardson alpha coefficient 0.49 to 0.77 and for inter-rater reliability, agreement % = 0.78 to 1.0)^15^.

A repercussion index was calculated for each hallucination by adding the score obtained at the quantitative section of the PSAS: frequency (Fq), duration (Du), unpleasant or negative aspects (NA), conviction (C), impact (I), control (Ctrl) and sound intensity (SI) (the latter only for auditory hallucinations). Range of score was [0–27] for auditory hallucinations and [0–23] for visual, olfactory/gustatory and coenesthetic hallucinations.

### Collected data

The evaluations were performed by psychiatrists specialized in the evaluation of SCZ patients (ICB, GB, DM, AMT, RS, CL, PML) and neurologists specialized in the evaluation of PD patients (GF, AM, BD, FD). A training session using a questionnaire-based diagnostic guidelines was conducted to reduce variability on rating.

Socio-demographic and therapeutic clinical data were collected for all of the participants during the interview for the administration of the PSAS.

The use of dopaminergic agonists, amantadine, anticholinergics, psychoactive drugs (antidepressants, antipsychotics including clozapine, anxiolytics and/or hypnotics) was recorded. The levodopa equivalent daily dose and chlorpromazine equivalent daily dose were calculated using published and validated equivalence schemes^16^,^17^.

### Inclusion and exclusion criteria

Inclusion criteria were:

- Participants older than 18 years of age.

- For the SCZ group: schizophrenia, according to DSM-IV-TR diagnosis criteria including a positive score criterion for hallucination (A2: characteristic symptoms) and a score up to three for the ‘hallucinatory behavior’ item of the *Positive and Negative Syndrome Scale* (PANSS P3)^18^.

- For the PD group: a diagnosis of Parkinson’s disease based on the UK Brain Bank criteria^19^ and a score above one for the ‘thought disorder’ item of the modified version of *Unified Parkinson’s Disease Rating Scale part 1* (UPDRS1 I2)^20^ (‘delusions’ and ‘florid psychosis’ terms have been deleted).

Exclusion criteria were:

- An inability to understand the instructions because of language or an underlying severe pathology.

- Mini Mental State Examination (MMSE) < 24.

- A primary diagnosis of any psychiatric disorder Axis I (including Substance-related Disorder, Mood Disorder and Anxiety Disorder), apart from schizophrenia in the SCZ group.

### Ethics

The study was conducted according to good clinical practice. This research has been evaluated and qualified by Ethics Committee (Committees of Protection of Persons Sud Est 6) as non-interventional clinical trial in accordance with French law that requires only a free, informed form. Written consent is not required in this case. CHU Clermont-Ferrand –FRANCE approved the experimental protocol.

### Data Analysis

Statistical analysis was performed using Stata software, version 13 (StataCorp, College Station, TX, U.S.). The tests were two-sided, with a type I error set at α = 0.05. Continuous data were presented as the mean ± standard deviation or the median [interquartile range] according to statistical distribution (assumption of normality checked using normal probability plots and Shapiro-Wilk’s test). Comparisons between independent groups were analyzed using the Student t-test or Mann-Whitney test when conditions of the t-test were not met (normality and homoscedasticity verified by the Fisher-Snedecor test) for quantitative variables. Comparisons concerning categorical data were performed using the Chi-Squared test or Fisher’s exact test. For each pathology (SCZ and PD), a Venn diagram^21^ was proposed to illustrate the relationships between the dimensions of PSAS. More precisely, a five-set Venn diagram using congruent ellipses in a 5-fold rotationally symmetrical arrangement devised by Grünbaum was performed. A Venn diagram is constructed with a collection of simple closed curves drawn in a plane. The principle of these diagrams is that classes be represented by regions in such relation to one another that all the possible logical relations of these classes can be indicated in the same diagram. Venn diagrams comprise overlapping circles. The interior of the circle symbolically represents the elements of the set, while the exterior represents elements that are not members of the set. Venn diagrams do not generally contain information on the relative or absolute sizes of sets; i.e. they are schematic diagrams.

## Results

### Characteristics of the population

Socio-demographic and clinical characteristics are summarized in Table 1. The study included 100 PD and 100 SCZ patients (86 considered as early onset patients and 14 considered as late-onset according to the criteria defined by Howard *et al*.^22^). There was no difference between the two groups in regards to anxiolytics, antidepressant drug or sedative-hypnotic drug ratios.

### Prevalence and associations between different hallucinatory experiences

Hallucination occurrences in every modality are summarized in Table 2. Each hallucination sensory modality significantly differed between PD and SCZ patients. The highest prevalence rate for “guardian angel” syndrome was observed in PD even though this syndrome was reported in half of the SCZ sample. Auditory, olfactory/gustatory and coenesthetic hallucinations were more frequent in SCZ compared to visual hallucinations which were more prevalent in PD. The combination of auditory and visual hallucinations was the most frequent for both PD and SCZ, but the distribution of the total number of sensory modalities significantly differed between the two groups.

**Figure 1:**
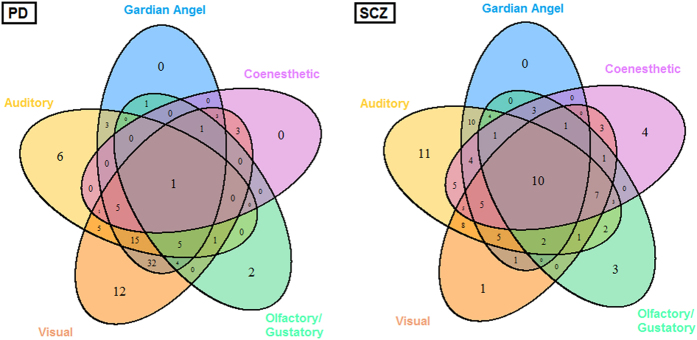
Venn diagrams showing intersections between hallucination sensory modalities in Parkinson’s disease (PD) and schizophrenia (SCZ) patients (n = 200). Understanding results guide For PD patients, 32 = Visual ∪Auditory ∩ Guardian angel ∪ Coenesthetic ∪ Olfactory For SCZ patients, 7 = Visual ∩ Auditory ∪ Guardian angel ∩ Coenesthetic ∩ Olfactory.

Combinations of hallucination sets (Fig. 1) seemed specific for each disease. For SCZ the distribution of subsets of hallucinations was nearly well balanced. On the contrary, visual hallucinations predominated over mono- or multi-modalities in PD. For the two disorders, the guardian angel syndrome was always found combined with at least one other hallucination sensory modality. There’s no significant correlation between L-dopa equivalent daily dose or chlorpromazine equivalent daily dose and number of hallucinations.

### Repercussion index

Regarding the quantitative evaluation of hallucinations, only the repercussion index for olfactory/gustatory hallucination did not significantly differ between the groups (Table 3). For the other sensory modalities involved in hallucinations, the repercussion index varied significantly across groups, with the highest mean score in the SCZ group. Auditory hallucinations exhibited the highest repercussion index among sensory modalities, for both PD and SCZ groups. L-dopa equivalent daily dose was not significantly correlated with repercussion index of each sensory modalities. Chlorpromazine equivalent daily dose was only significantly and weakly correlated with auditory hallucinations repercussion index (r = 0.26, p = 0.01).

**Figure 2:**
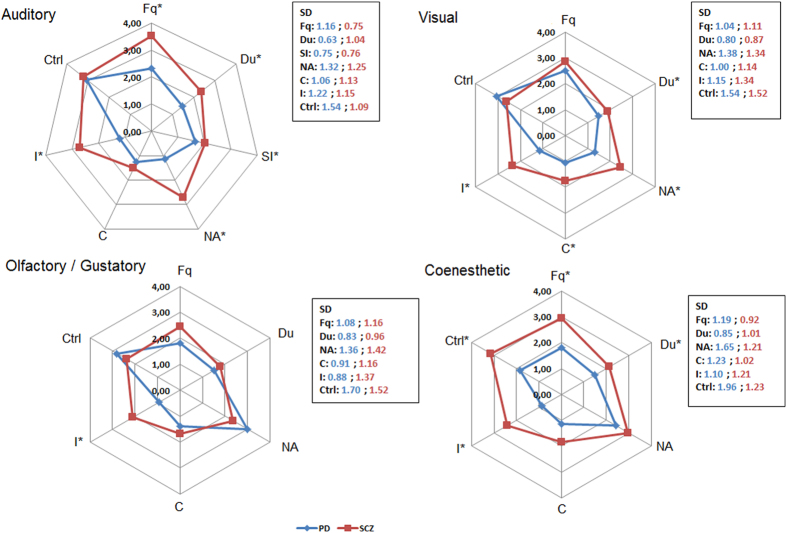
Spider diagrams of repercussion factors of hallucinations in Parkinson’s disease (PD) and schizophrenia (SCZ) patients. Frequency (Fq), duration (Du), unpleasant or negative aspects (NA), conviction (C), impact (I), control (Ctrl) and sound intensity (SI). For each spider diagram, data are expressed as means and standard deviations (SD) were placed in a box. *p < 0.05 (comparison between pathologic groups).

Quantitative evaluations of hallucinations are detailed in Fig. 2. The frequency of hallucinations was significantly higher for SCZ patients in the auditory and coenesthetic modalities. The duration of hallucinations was also significantly higher for SCZ in the auditory, visual and coenesthetic modalities. Negative contents were significantly more frequently reported by SCZ subjects in auditory and visual hallucinations. Conviction was found higher for SCZ only for visual hallucinations, while the feeling of being in control was higher in the same subjects only for coenesthetic experiences. Finally, the sound intensity of auditory hallucinations was higher for SCZ compared with PD patients.

## Discussion

To our knowledge, the present study is the first to qualitatively and quantitatively describe the phenomenological properties of hallucinatory experiences, across sensory modalities and categorical diagnoses (i.e. SCZ and PD). The main results can be summarized as follows:

- In SCZ, auditory hallucinations were observed in 83% of the cases, but visual and coenesthetic hallucinations were also reported by more than 50% of patients. Different sensory modalities were combined in 81% of the patients, and within 55% of the cases two or three modalities were combined. In PD, visual hallucinations appeared predominant (88%), but 42% of the patients also described auditory experiences. In our study population, 80% of the PD patients described more than one sensory modality involved, with 44% associating two modalities (most frequently visual and auditory).
- The guardian angel item, which is usually not explored in SCZ, was described by 46% of SCZ patients.
- The repercussion index, created to describe the impact of hallucinations in the patients (i.e. this index gathered the scores for frequency, duration, intensity, negative content, beliefs and perceived control), demonstrated that SCZ patients were more affected by hallucinations compared with PD patients.
- Except for the olfactory and gustatory sensory modalities (which differed only in terms of impact), SCZ and PD significantly differed in terms of hallucination properties: i.e. frequency, duration, capacity of control, negative valence and impact.

Rates of hallucinatory experiences in the different sensory modalities appeared consistent with the literature both for SCZ^23^ and non-demented treated PD patients^24^. In our study, a major finding was the high rate of simultaneous multimodal hallucinations, which are poorly described in SCZ patients despite the fact that they are considered to be rather frequent in the non-clinical population^25^. A recent internet survey was conducted in the general population (n = 153) to phenomenologically explore auditory hallucinations^26^. The authors showed that 28% of the participants (n = 43) reported an association of auditory hallucinations with “distinct hallucinations in other senses”. Thirty-seven of the participants (24%) were diagnosed as SCZ or schizoaffective patients, and none were described as PD patients. No specific information was given about the multimodal expression of hallucinations in this sub-sample. The lower percentage of association of various modalities of hallucinations compared to our study can be related to the different study population and to differences in the method of hallucination evaluation.

In the literature^22^,^27^, late-onset schizophrenic patients are considered to complain more of visual, tactile and olfactory hallucinations, third-person running commentary, and accusatory or abusive auditory hallucinations, than early-onset patients. In our sample, if late-onset patients present more hallucinations with a smaller level of repercussion, compared to early-onset patients, these differences don’t reach statistical significance (respectively p = 0.06 and p = 0.08). This lack of difference can be related to the small size of our sample, and will have to be further studied.

For PD, our results are consistent with the findings of Goetz *et al*.^9^. These authors demonstrated that non-visual hallucinations emerged over time: after a ten-year observation period of 60 PD patients, 60% had multimodal hallucination symptomatology. In this case, the question arises whether dementia can have an impact but previous multivariate analysis^28^ and some univariate works^29^ already show that cognition impairment did not correlate as an independent variable significantly with Parkinson disease duration In our sample, the mean duration of PD was 10.76 years (SD 5.8), which is comparable with the study by Goetz *et al*. (9.0 ± 6.2 years).

Our two samples appear to be quite representative of SCZ and PD and haven’t any other clinical or treatment specificity compared to what was described in the literature that could be correlated to this observation. This result exemplifies the potential of the PSAS to systematically explore hallucinations in every modality.

The prevalence of “presence hallucinations”, described by the “guardian angel” item of the PSAS, in PD is rather well-known, ranging from 34 to 40% in samples of consecutively examined patients^10^. In the present study, the percentage was higher (70%). This difference can be related to the specific inclusion of hallucinating PD patients in our study group, but also to the systematic identification of such experiences using the PSAS.

It’s noteworthy that, despite this high percentage of presence hallucinations, the mean L-dopa equivalent daily dose (LEDD) was very close to the one reported by Fenelon *et al*.^10^. (876 mg/d, SD 573 *vs* 892 mg/d, SD 389). The mean LEDD in the sub-group of patients (n = 70) with presence hallucinations was 864 (SD 564) mg/d.

Jaspers described this phenomenon in 1913, in patients suffering from dementia praecox (i.e. schizophrenia), under the name of “*leibhatige Bewussheit*”, which has been translated as “sense of presence”, but also ‘idea of presence’: “*There are patients who have a certain feeling (in the mental sense) or awareness that someone is close by, behind them or above them, someone that they can in no way perceive with the external senses, yet whose actual/concrete presence is directly/clearly experienced*” (translated in Koehler and Sauer, 1984^30^). The discussion about the fact that it can be considered as a hallucination or a delusion can be related to the difficulty to operationalize in clinical practice the notions of “feeling in a mental way or awareness”^30^,^31^. In the DSM-5 criteria for schizophrenia, the “*sense of presence of an unseen person*” is considered to be one of the “*unusual perceptive experiences*”, part of the continuous disturbances described in the Criterion C (p 101). Presence hallucinations can be considered as residual symptoms. In our SCZ sample, the percentage of patients describing this experience is rather high (46%), but to our knowledge, there are no other studies to compare this level with. It probably deserves a more specific phenomenological evaluation in future studies.

By creating a composite index combining frequency, duration, intensity, negative content, beliefs and perceived control, we were able to describe in a reproducible manner the impact of hallucinations on patients. In our study, visual, auditory and coenesthetic hallucinations exhibited greater impacts on SCZ patients compared with PD patients. This can notably be correlated to the negative impact of hallucinations on SCZ patients in terms of quality of life and ability to function^32^. Nevertheless, the impact of positive symptoms on quality of life appears relatively weak in individuals in the early course of the illness compared to chronic patients^33^. The mean duration of illness in the sub-group of SCZ patients is 8.1 years (SD 6.8), and they can’t be considered to be in an early stage of schizophrenia. Specific interventions integrating coping training and cognitive-behavioral therapy and focusing on hallucinations induced a significant improvement in quality of life and ability to function^34^. On the other hand in PD patients, hallucinations do not seem to be a major predictor of impaired quality of life regardless cognitive profile^35^,^36^. Depression has the most negative influence on ability to function in those patients.

### Perspectives

Conceptually, the present study, based on the PSAS, confirmed the possibility of exploring hallucinations transdiagnostically as recently proposed through the RDoC initiative. Indeed, to move beyond strict categorical diagnoses, it appears crucial to determine if hallucinations are the same phenomena in different clinical populations^37^. This is called the “equifinality” purpose in the RDoC framework^6^. The fact that SCZ and PD patients may share common hallucinatory experiences, but with different phenomenological properties (i.e. potentially linked with different RDoC domains) clearly raises new questions regarding their possible mechanisms. The present study is not able to answer such a complex question but the availability of transdiagnostic tools and data are a necessary starting point. The idea behind the RDoC domains is the possibility to link specific phenomenological features with cognitive or neurobiological mechanisms. Few studies have explored the neural basis of the phenomenological properties of hallucinations, as for example: the sense of reality^38^, the sensory modalities involved^39^, or the emotional content^40^. The next step is to combine these detailed studies of hallucination phenomenology with transdiagnostic approaches. We believe that tools such as the PSAS could help design such experiments in the future.

### Strengths

To our knowledge, this work is the first to explore the hallucination phenomenon in a multimodal way, in two different clinical samples and using a specifically designed tool. These three points are the principal strengths of our study. This study analyzed patients with SCZ and PD, two illnesses in which hallucinations can be frequent, and are related to a dopaminergic dysfunction. The rather large size of our two sample populations, compared to previously published studies, can also be considered a strength as it allowed comparison of the different modalities of hallucinations between the two groups. The description of hallucination impact on patients, using the repercussion index, could be of interest to define individualized treatment goals for patients.

### Limitations

One of the limitations of this study is the cross-sectional design, which doesn’t allow for study of the evolution of hallucinations over time or their relations, which is of interest as underlined by different authors^41^. The use of a structured tool can be considered another limitation to this study, as the previously made assumptions about the nature of hallucinations that were made to build this evaluation scale may limit the phenomenological description^26^.

## Conclusion

The phenomenological specificity of hallucinations regarding pathologies can contribute significantly towards their comprehension, and ongoing publications^26^,^42^,^43^ show promising results. A broader view including widespread characteristics, such as age, gender, cognitive deficits or treatments and interdisciplinary works, must be encouraged to better understand the complexity of the process involved in hallucinations.

## Additional Information

**How to cite this article**: Llorca, P. M. *et al*. Hallucinations in schizophrenia and Parkinson’s disease: an analysis of sensory modalities involved and the repercussion on patients. *Sci. Rep.* **6**, 38152; doi: 10.1038/srep38152 (2016).

**Publisher's note:** Springer Nature remains neutral with regard to jurisdictional claims in published maps and institutional affiliations.

## Figures and Tables

**Table 1:** Sociodemographic and medical details of Parkinson’s disease (PD) and schizophrenia (SCZ) patients (n = 200).

	PD (n = 100)	SCZ (n = 100)	p
Sex – Male/Female (no)	55/45	69/31	**0.04**
Age (y): mean (SD)	71.1 (7.7)	36.5 (11.6)	**<0.001**
Disease duration (y): mean (SD)	10.8 (5.9)	8.2 6.8)	**<0.001**
H&Y stage: mean (SD)			
Treatments (no)	2.8 (0.9)	NA	NA
Antipsychotics			
0	88	0	**<0.001**
1	12	52	
2 different antipsychotics	0	35	
3 different antipsychotics	0	13	
Antidepressants	18	17	0.91
Anticholinergics	8	25	**0.001**
Anxiolytics			
0	86	74	**0.05**
1	14	25	
2 different	0	3	
Sedative-hypnotic drugs	5	10	0.2
Levodopa	98	0	NA
Dopamine agonists	39	0	NA
Chlorpromazine equivalent daily dose: median [IQR]	NA	658 [400–1125]	
LEDD: median [IQR]	690 [500–1093]	NA	

No: number, y: years, H&Y stage: Hoehn and Yahr stage^44^, LEDD: Levodopa Equivalent Daily Dose NA: Not Appropriate.

**Table 2:** Hallucination profile for Parkinson’s disease (PD) and schizophrenia (SCZ) patients (n = 200).

	Number of PD (n = 100)	Number of SCZ (n = 100)	p
Guardian angel syndrome	70	46	**<0.001**
Auditory hallucination	45	83	**<0.001**
Visual hallucination	88	55	**<0.001**
Olfactory/gustatory hallucination	15	38	**<0.001**
Coenesthetic hallucination	14	52	**<0.001**
Number of combined hallucination(s)			
1	20	19	0.86
2	44	29	**0.03**
3	24	26	0.74
4	11	16	0.30
5	1	10	**0.01**

**Table 3:** Repercussions of hallucinations in Parkinson’s disease (PD) and schizophrenia (SCZ) patients (n = 200).

Indices of repercussion# of:	PD	SCZ	p
Auditory hallucination, mean (SD)	12.1 (4.9)	18.0 (3.6)	**<0.001**
Visual hallucination, mean (SD)	10.6 (4.6)	14.0 (4.0)	**<0.001**
Olfactory/gustatory hallucination, mean (SD)	11.5 (4.9)	12.8 (3.7)	0.36
Coenesthetic hallucination, mean (SD)	9.6 (5.7)	15.5 (3.5)	**0.002**

^#^Range of score for auditory hallucination: [0–27]; for visual, olfactory/gustatory and coenesthetic hallucination: [0–23].
